# Ultrasound-Guided Percutaneous Drainage of Abdominal Abscess in a Patient With Crohn's Disease: A Case Report

**DOI:** 10.3389/fsurg.2021.616586

**Published:** 2021-06-04

**Authors:** Radmila V. Karpova, Ksenia S. Russkova, Roman N. Komarov, Arina A. Petrova

**Affiliations:** ^1^Department of Faculty Surgery No. 1, University Clinical Hospital No. 1, I.M. Sechenov First Moscow State Medical University (Sechenov University), Moscow, Russia; ^2^Institute of Clinical Medicine, I.M. Sechenov First Moscow State Medical University (Sechenov University), Moscow, Russia; ^3^International School “Medicine of the Future”, I.M. Sechenov First Moscow State Medical University (Sechenov University), Moscow, Russia

**Keywords:** abdominal abcess, percutaneous drainage, ultrasound, immunomodulatory therapy, Crohn's disease

## Abstract

**Introduction:** The autoimmune process in Crohn's disease exacerbates destructive changes in the intestinal wall and leads to complications such as bleeding (21. 9%), strictures (21.6%), and abscesses (19.7%).

**Case Presentation:** The case of a 32-year-old male patient with an 8-year history of Crohn's disease is presented. He was admitted for emergency indications with severe pain in the right lower quadrant, chills, and a fever reaching 39.0°C. The patient had anemia, hypocoagulation and immunodeficiency. Ultrasound and CT scans of the abdominal organs revealed an abscess in the right iliac region. It was immediately drained under ultrasound control and X-ray. A fistulogram showed the fistula between the abscess and the ileum. Routine antibiotic therapy selected in accordance with the sensitivity of the microflora and sanitization of the abscess cavity were not effective. The immunomodulatory therapy, intravenous administration of cryoprecipitate, and the introduction of fibrin glue into the abscess cavity were added to the treatment. After the treatment, the patient's immune status corresponded to normal, the abscess healed, and the fistula was closed.

**Conclusion:** In patients suffering from Crohn's disease with the formation of an abscess and a long-term non-healing intestinal fistula, it is essential that the diagnostic algorithm includes the examination of the immune status. Treatment should include immunomodulators, intravenous administration of cryoprecipitate. To close the fistula in these patients, it is advisable to use fibrin glue that has a local immunomodulatory effect.

## Introduction

The reasons behind the chronic intestinal inflammatory process in Crohn's disease (CD) are still the subject of discussion. The prevailing opinion is that Crohn's lesions result from abnormalities in components of innate immunity, accompanied by changes in the differentiation and activation of T- and B-lymphocytes (1, 2). Other studies show that mild and moderate forms of CD are mostly associated with immune activation, while severe cases are characterized by immunodeficiency that aggravates the destructive process in the gastrointestinal tract and leads to the development of bleeding (24.7%), the formation of strictures (21.6%), and abscesses (19.7%) (3).

## Case Presentation

A 32-year-old male patient with an 8-year history of CD was admitted for emergency indications to the Department of Faculty Surgery №1 of the First Moscow State Medical University (Sechenov University) with severe abdominal pain, chills, weakness, and fever reaching 39.0°C. According to the patient's anamnesis, he had been taking hormone therapy (budesonide 18 mg/day) for 5 years and there were 3 episodes of abscesses without fistulas, which were percutaneously drained under ultrasound control. He denied allergic reactions, use of alcohol, cigarettes. His family history was not significant.

**Figure 1:**
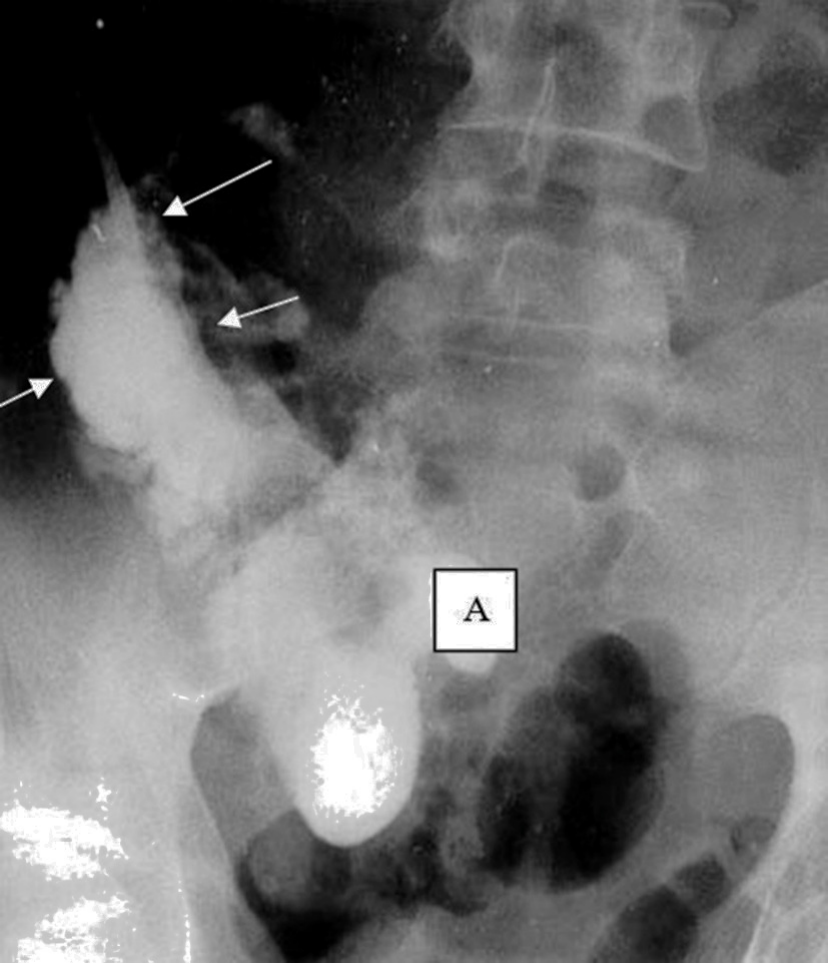
Ultrasound image of the abdominal abscess (arrows) and a sequestrum (A).

**Figure 2:**
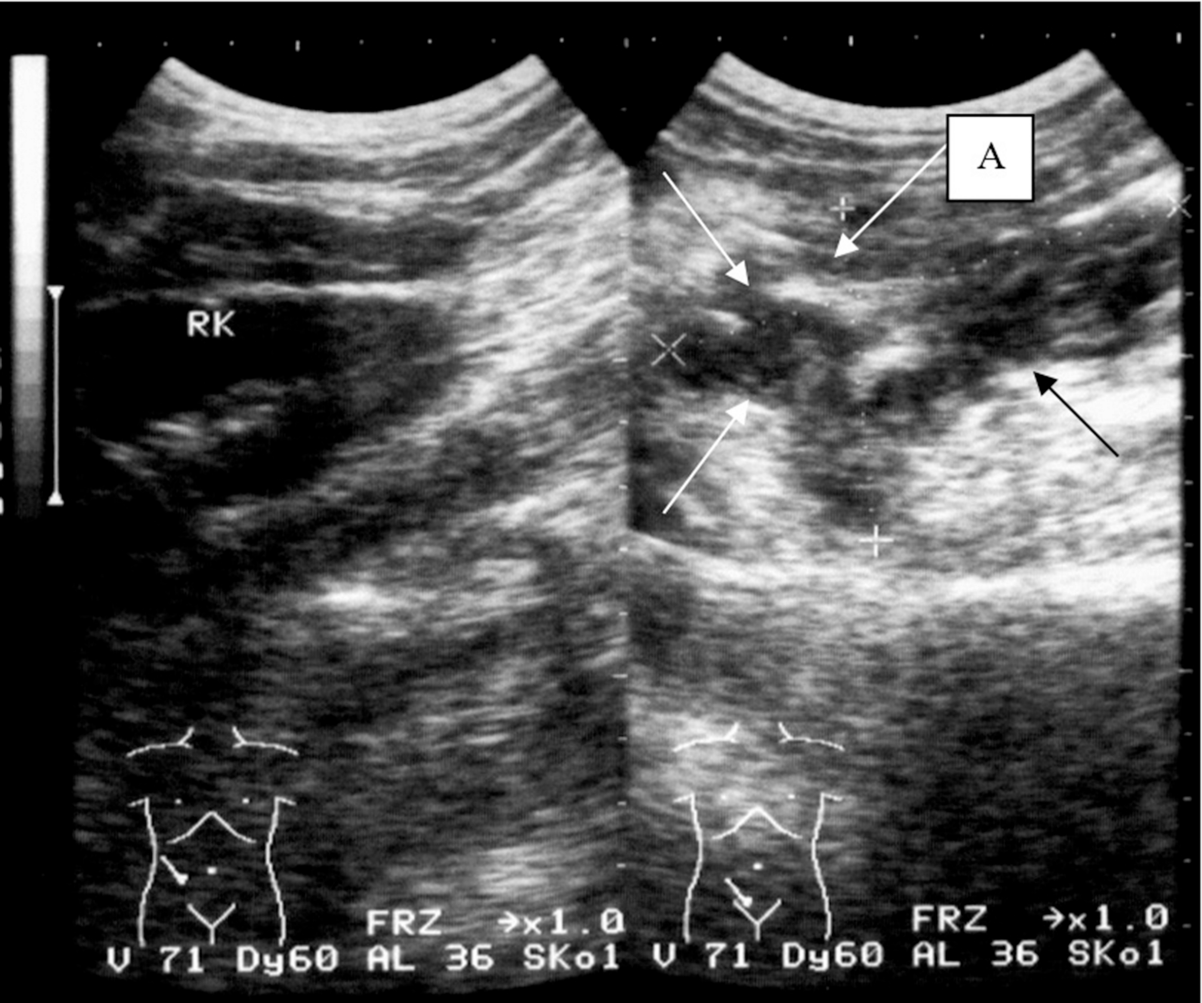
X-ray image of the abdominal abscess in the right iliac region (arrows) and the ileocecal angle of the intestine (A).

The symptoms developed acutely during the day. The patient was admitted to the Sechenov University for surgical treatment. At admission, the condition was severe, and the patient's posture was visibly contorted due to pain in the lower abdomen. The lungs exhibited vesicular respiration and no wheezing. The blood pressure was 100/80 mm Hg. The pulse rate was 92 beats per minute. The abdomen was painful on the right side and positive peritoneal symptoms were exhibited. In the blood test, red blood cell count was 4.4 × 10^12^ cells/L, hemoglobin was 112 g/L, the leukocyte (white blood cell) count was 19 × 10^9^/L, the erythrocyte sedimentation rate was 16 mm/h, and the C-reactive protein reading was 21.0 mg/dL. Ultrasound and CT of the abdominal cavity revealed a 110 mL abscess in the right iliac region (Figure 1). With the patient under local anesthesia, the abscess was immediately percutaneously drained under ultrasound control. One-hundred ml of pus was obtained and sent to for microbiological investigation. The fistulogram with contrast (Omnipaque 76%) showed a cavity with uneven, indistinct contours and heterogeneous content in the right lower quadrant (volume 10 × 6 cm), and the contrast flowed into the terminal ileum (Figure 2). The abscess cavity was irrigated using antiseptic solution. The outflow drain was left in place.

## Intraoperative Diagnosis

The patient had an 8-years history of CD with previous episodes of abscesses; the follow-up data led to the diagnosis accurately. The patient was diagnosed with a severe form of CD, exhibiting terminal ileitis, a chronic relapsing course, and an abdominal abscess in the right lower quadrant communicating with the ileum.

Sanitization of the abscess cavity with antibiotic solutions (ciprofloxacin and metronidazole) selected in accordance with the sensitivity of the microflora was performed (daily for 10 days). Despite this, there was no positive change; the fistula did not heal.

Immunological examination of the patient revealed a decreased immunoregulatory index, corresponding to 0.8 (the normal range is 1.0–2.0), and the activation of monocyte/macrophage cytokines (interleukin-1, interleukin-6, tumor necrosis factor α, etc.) that is common in severe courses of CD (4). Based on the obtained data, the patient was administered immunomodulatory therapy (intravenous immunoglobulin 10 mg/day for 5 days). Inside the abscess cavity, fibrin glue “KRIOFIT” (Patent RUS №110991, 4.02.2011) was injected (5 ml with exposure for 2 h, after washing the abscess, once a day for 7 days). Fibrin glue consists of cryoprecipitate and thrombin and also has a local immunomodulatory effect. Besides that, the patient had hypofibrinogenemia and hypocoagulation (prothrombin index 65%, platelets 120 × 109/L, fibrinogen 1.6 mg/mL), so a 7-day course of intravenous cryoprecipitate was administered. Hormone therapy was discontinued.

Due to the treatment, the patient's condition improved, the fistula was closed on the seventh day, and the immunoregulatory index corresponded to normal. The coagulopathy was compensated. The length of hospital stay was set to 21 bed-days.

Currently, hormone therapy is not performed, and the patient receives immunomodulatory therapy (imunofan) and sulfasalazine 1.5 mg/day. He expressed his gratitude to the surgeons who managed to help him and made him comfortable. Every 6 months, a dynamic follow-up was conducted, and no negative dynamics were detected.

## Discussion

The majority of researchers believe that the use of immunomodulators is a promising approach to maintaining remission in CD (5, 6). According to previous reports, the B-cell immune response plays an important role in fistula formation, realized through the secretion of immunoglobulins and their destructive influence on the intestinal wall (7). During this process, bacterial pathogens penetrate through the epithelial barrier and stimulate components of the mucosal immune system (8–10). As a result of the destruction and perforation of the intestinal wall due to long-term hormone therapy, the patient developed an abdominal abscess, that was percutaneously drained under the control of ultrasound.

It has been proven that cryoprecipitate has not only hemostatic but also local and systemic anti-inflammatory and immunomodulatory effects due to pro- and anti-inflammatory cytokines in its composition (spontaneous interferon, interleukin-2, interleukin-8, plasminogen, albumin, globulin fraction etc.) (11). Fibrin glue that was injected into the abscess cavity also has a local immunomodulatory effect (12). The literature considers that the abscess communicating with the bowel loop can heal on its own (13). In our case, the patient with CD had an abdominal abscess that communicated with the ileum as a complication. However, in this case, due to the patient's severe immunodeficiency and hypocoagulation as a result of hormone therapy and nutritional deficiency, the fistula between the abscess and the ileum did not heal for 10 days, despite receiving therapy according to the standards of medical care. Peritonitis and sepsis could occur.

Performing immunomodulatory therapy, discontinued hormone therapy, intravenous administration of cryoprecipitate, and the introduction of fibrin glue into the abscess cavity (that is not currently included in the standards of medical care in our institution) together with routine antibacterial therapy enabled the normalization of general and local immunity, closure of the fistula, and healing of the abscess for 7 days.

## Conclusion

In patients suffering from CD with the formation of an abscess and a long-term non-healing intestinal fistula, it is essential that the diagnostic algorithm includes the examination of the immune status. Treatment should include the immunomodulatory intravenous administration of cryoprecipitate. To close the fistula in these patients, it is advisable to use fibrin glue that has a local immunomodulatory effect.

## Data Availability Statement

The original contributions presented in the study are included in the article/supplementary material, further inquiries can be directed to the corresponding author/s.

## Ethics Statement

Written informed consent was obtained from the individual(s) for the publication of any potentially identifiable images or data included in this article.

## Author Contributions

RVK, KSR, RNK, and AAP contributed to the manuscript equally, from conception and design, administrative support, provision of study materials or patients, collection and assembly of data, data analysis and interpretation, manuscript writing, and final approval of the manuscript.

## Conflict of Interest

The authors declare that the research was conducted in the absence of any commercial or financial relationships that could be construed as a potential conflict of interest.
